# Ten Years of Universal Testing: How the Rapid Diagnostic Test Became a Game Changer for Malaria Case Management and Improved Disease Reporting

**DOI:** 10.4269/ajtmh.21-0643

**Published:** 2021-11-08

**Authors:** Michael Aidoo, Sandra Incardona

**Affiliations:** ^1^ Division of Parasitic Diseases and Malaria, Centers for Disease Control and Prevention, Atlanta, Georgia;; ^2^ Foundation for Innovative New Diagnostics, Geneva, Switzerland

## Abstract

In 2010, the World Health Organization changed its guidance on malaria case management, recommending parasitological confirmation of all suspected cases before treatment with an antimalarial. This recommendation was in large part as a result of the availability of quality assured malaria rapid diagnostic tests (RDTs) that made it possible for malaria diagnosis to be performed by laboratory staff in all health facilities irrespective of the facility’s place in the tiered health system. Community health workers and other non-laboratory health workers who traditionally did not perform malaria testing due to the technical and logistic demands of smear microscopy were now able to test for malaria. The use of RDTs has led to substantial increases in testing rates, improved quality of case management, as well as more accurate reporting of malaria cases. Although current RDTs have limitations, they remain one of the most important tools in contemporary malaria control. Further improvements to existing products, such as increased sensitivity for non-falciparum tests, diversification of *Plasmodium falciparum* antigen targets, along with strengthened health system support for current RDTs will further enhance their utility in malaria control and prevention.

## INTRODUCTION

Since 2010, the World Health Organization (WHO) recommends universal testing of all suspected malaria cases with a quality assured test before treatment.^1^ This change was in part prompted by reductions in malaria incidence in most endemic countries, making the often-used clinical diagnosis based on fever alone even more non-specific, and by the availability of quality assured malaria rapid diagnostic tests (RDTs). These RDTs allowed testing in different types of settings outside laboratories including by community health workers (CHWs). RDTs also helped address high workloads when microscopy was used and the general lack of quality microscopy (availability and competency) across most endemic countries.^2^^,^^3^

The WHO together with U.S. Centers for Disease Control and Prevention (CDC), the Foundation for Innovative New Diagnostics, the Global Fund to Fight HIV, Tuberculosis and Malaria, the U.S. President’s Malaria Initiative, and other organizations guided RDT implementation by providing multiple resources including guidance for procurement, storage and transport, training materials, and tools for quality assurance.^4^ Over the last decade, the availability of these simple but reliable tests has led to major changes in several aspects of malaria control, including surveillance, increased testing of suspect cases, and improved case management of febrile illness at health facility, and in the patients’ homes by CHWs.

## QUALITY ASSURED RDTS

First-generation RDTs were often of low or unknown quality.^5^ The WHO, in collaboration with global partners, instituted a malaria RDT product testing program that consisted of a comparative performance evaluation of RDTs on the market,^6^ and formed the basis of WHO’s product selection criteria. In addition, a pre-procurement lot testing program checked the quality of each RDT lot procured by major donor organizations as well as country programs.^7^ These two activities ensured that RDTs used in endemic countries were of good quality and led to an overall improvement in RDT performance, with the percentage of RDT products meeting WHO procurement criteria increasing from 26.8% to 79.4% between 2008 and 2018.^6^^,^^7^ RDT performance evaluation is now incorporated into the WHO Pre-Qualification of in vitro diagnostics scheme and pre-procurement lot testing is currently not performed on all procurements but rather on an ad-hoc basis based on procurers’ policies and practices.

## INCREASED TEST RATE AND ADHERENCE TO TEST RESULTS

**Figure 1.**
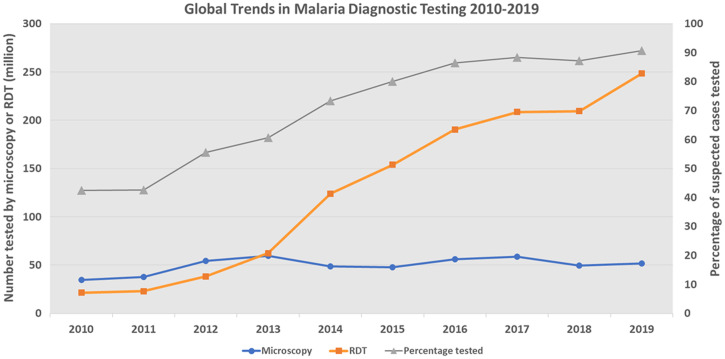
Global trends in malaria diagnostic testing 2010–2019. This figure appears in color at www.ajtmh.org.

Testing rates for suspected malaria cases increased from 36% in 2010 to over 84% in 2018 in sub-Saharan-Africa (SSA) (Figure 1). Most of the increase was attributed to increased use of RDTs, which accounted for 60–80% of all malaria diagnosis from 2013 to 2019.^8^ Approximately 2.7 billion malaria RDTs were supplied by manufacturers from 2010 to 2019, with an increase from less than 100 million in 2010 to approximately 348 million in 2019.

An initially low adherence to RDT test results led to high rates of malaria negative cases being unnecessarily treated with antimalarials, partly because of relatively low test positivity rates when compared with imprecise clinical diagnosis and high false positivity rates in settings with low-quality microscopy.^9^^,^^10^ The trust in RDTs increased as evidence of the inadequacy of these other methods began to accumulate, in parallel with more frequent studies exploring the true causes of fever,^11^ and increased awareness that RDT quality was verified via the product and lot testing programs.

## INCREASED ACCESS TO TESTING THROUGH RDT USE IN COMMUNITIES

One of the most important benefits of malaria RDTs has been their use in communities typically underserved by routine health services. CHWs, who are often volunteers trained to offer certain basic health services, now routinely test for malaria using RDTs and treat patients based on the test result.^12^^,^^13^ Malaria is currently the only major infectious disease routinely tested for by CHWs as part of integrated community case management.^14^ With supplies of malaria tests, antimalarial drugs, oral rehydration salts, and zinc, CHWs are able to offer to an extent differential management of febrile disease in the community thus improving access to healthcare.^15^

## RDT USE IN THE FORMAL PUBLIC HEALTH SECTOR

Malaria RDTs allow reductions in turnaround times in larger health facilities with laboratories, especially in high burden malaria regions, where a combination of patient load and inadequate staffing levels for microscopy can sometimes overwhelm laboratories.^16^ In some countries, outpatient nurses are able to test suspected malaria patients using RDTs before patients see clinicians.^16^ Even when microscopes are available, inadequate support for items such as reagents, supplies, equipment maintenance, and training to provide consistent high-quality testing make RDTs a better choice for malaria diagnosis in some settings.

## PRIVATE SECTOR RDT USE

RDT use in the private sector has not been as extensive as in the public sector, with challenges including the established behaviors of practice and the absence of a regulatory framework within which RDT use in the private sector can be promoted.^17^^,^^18^ However, evidence suggests that use of incentives and other market-based approaches as well as training, supervision, and quality assurance such as in Accredited Drug Dispensing Outlets, have the potential to improve coverage and quality of malaria case management services in these settings.^19^ A roadmap to guide countries seeking to expand such efforts has been published by the WHO and global partners.^20^

## IMPACT AND CONSEQUENCES OF INCREASED RDT USE

The impact of testing became apparent early during RDT roll-out with some countries demonstrating reductions in antimalarial drug consumption as treatment was increasingly guided by test results rather than by fever alone.^21^^,^^22^ Such an impact was achieved particularly in settings where reliance on RDT results for treatment decisions was high. Efforts to sustain adherence and consistent testing practices continue as RDTs are rolled out at larger scale and in more challenging settings such as in communities and the private sector. The increased use of malaria RDTs has also led to increased awareness of non-malarial febrile illnesses, which were found at higher proportions than expected. Unnecessarily high use of antibiotics to treat malaria-negative patients who test negative with malaria RDTs has also been reported.^11^^,^^23^ This calls for larger scale RDT implementation to be accompanied by testing for other diseases and collaboration across health sectors to strengthen management of febrile illness.

## IMPROVING RDT PERFORMANCE, AVAILABILITY, AND USE

Further improvements for increased impact are still needed, with challenges falling into two broad categories: technical and health systems.

### Technical challenges.

*Plasmodium falciparum*–specific Histidine Rich Protein- 2(HRP2)–based RDTs are more sensitive than tests based on targets such as *Plasmodium* lactate dehydrogenase (pLDH) in detecting *P. falciparum*, which is responsible for > 90% of malaria cases in SSA. One important threat to their efficient use for control and elimination of this malaria species is the existence of *hrp2/3*-deleted *P. falciparum* parasites that produce false negative HRP2 RDT results, with highest prevalence found in the Amazon region of South America.^24^^,^^25^ Prevalence of *hrp2/3*-deleted *P. falciparum* remains low in most of SSA. However, high prevalence has been reported in Eritrea and other countries in the Horn of Africa.^26^ Solutions to this problem include improvement of existing *P. falciparum*–specific pLDH and pan-LDH tests either as single targets or in combination with HRP2, along with development of tests based on new antigen targets that can be detected with increased sensitivity. Development of RDTs based on pLDH or other non-HRP2 targets could also solve the problem of HRP2 antigen persistence in blood for several weeks post treatment during which patients remain positive on HRP2-based but not pLDH-based RDTs.^27^ In high transmission settings, HRP2 persistence could skew accuracy of RDT-based surveys and challenges clinicians who must manage patients not knowing whether HRP2 RDT positivity is due to a recently cured or new infection.

Another technical challenge is that non-falciparum infections are less well detected than *P. falciparum* infections.^25^ Although less severe, non-falciparum mono-infections exist and cause disease in SSA, and are major causes of malaria in Southeast Asia, the Western Pacific Region, and South America. Improving the sensitivity of pLDH-based RDTs or identifying other targets would extend the utility of RDTs for more efficient control and elimination of all malaria species.

More generally, highly sensitive RDTs could represent one of several tools to support malaria elimination efforts, to better detect low-density parasitemia in asymptomatic individuals in approaches such as in screen-and-treat activities. Similarly, they could offer a better solution to detecting malaria in pregnant women with lower circulating parasitemia as a result of parasite sequestration in the placenta. A highly sensitive HRP2-based RDT is now commercially available and has been piloted in various studies,^28^^,^^29^ however, like the conventional HRP2-based RDT, this test will also produce false negative test results for infections caused by parasites with *hrp2/3* deletions. In addition, the lower recommended storage temperature and a shorter shelf life compared with conventional RDTs make their widespread use challenging. More targeted use cases therefore need to be identified until more robust versions of the test become available.

Another technical gap is the insufficient use of tools to monitor test quality under field settings. WHO international standards for *P. falciparum* are available from the United Kingdom’s National Institute for Biological Standards and Control, and dried parasite–infected blood preparations have been used as quality control material for assessment of health worker competence.^30^^,^^31^ Full use of these reagents remains to be realized, and post market surveillance is still weak for malaria diagnostics in endemic countries in general.

### Health systems challenges.

Health systems support for RDT use is highly variable. The quality of training for conducting the tests, supply chain management to avoid stock outs, troubleshooting in case of unusual results, and reporting of testing data especially from low level facilities and CHWs all need to be strengthened.

## CONCLUSION

A simple and inexpensive technology, the malaria RDT has revolutionized malaria control. The full potential of these RDTs is yet to be realized. There remain suspected malaria cases that do not receive a diagnostic test, and further scale-up of RDTs, including in the private sector, requires more attention. More sensitive pLDH or other non-HRP2 detecting RDTs are needed to facilitate diagnosis of infections with *hrp2*/3-deleted parasites, to address the challenge of HRP2 persistence, and to better detect non-falciparum infections. Many global partners came together to support the major investment required to roll out malaria RDTs. The remaining effort needed to optimize this existing technology should receive increased attention. The moment must be seized so that a similar story, maybe that of malaria elimination in many more regions, can be told in another 10 years with RDTs continuing to play a critical role.
